# Efficient Valorization of Waste Surgical Masks for the Production of Activated Carbon-like Sorbent and Its Application in Solid-Phase Extraction and UHPLC-PDA Analysis of Phthalates in Water

**DOI:** 10.3390/molecules31050877

**Published:** 2026-03-06

**Authors:** Pantaleone Bruni, Vanessa Da Fermo, Rafal Wolicki, Michele Ciulla, Pietro Di Profio, Leonardo Sbrascini, Francesco Nobili, Giuseppe Carlucci, Vincenzo Ferrone, Salvatore Genovese, Stefania Ferrari

**Affiliations:** 1 Department of Pharmacy, University “G. d’Annunzio” Chieti-Pescara, 66100 Chieti, Italy; pantaleone.bruni@unich.it (P.B.); vanessa.dafermo@unich.it (V.D.F.); rafal.wolicki@sentristech.com (R.W.); michele.ciulla@unich.it (M.C.); pietro.diprofio@unich.it (P.D.P.); giuseppe.carlucci@unich.it (G.C.); salvatore.genovese@unich.it (S.G.); stefania.ferrari@unich.it (S.F.); 2 UdA Tech Lab, University “G. d’Annunzio” Chieti-Pescara, 66100 Chieti, Italy; 3 Chemistry Division, School of Science and Technology, University of Camerino, Via Madonna Delle Carceri—ChIP, 62032 Camerino, Italy; leonardo.sbrascini@inl.int (L.S.); francesco.nobili@unicam.it (F.N.); 4 INL—International Iberian Nanotechnology Laboratory, Avenida Mestre José Veiga s/n, 4715-330 Braga, Portugal; 5 National Reference Center for Electrochemical Energy Storage (GISEL)-INSTM, Via G. Giusti 9, 50121 Firenze, Italy

**Keywords:** sample preparation, surgical mask upcycling, PAEs determination, UHPLC-PDA, waste valorization, characterization

## Abstract

One of the major current societal challenges concerns the reuse of waste materials and valuable substances to mitigate the environmental impact of human activities, which has led to the increasing release of pollutants, from plastics to pharmaceuticals. In this study, we report a simple recycling strategy for surgical masks to obtain an activated carbon-like material, suitable for the solid-phase extraction (SPE) of Phthalic acid esters (PAEss) from plastic bottled water. The sorbent was produced by high-temperature calcination after sulfuric acid treatment to enhance the thermal stability of polypropylene. The sorbent was characterized by thermal analysis, Raman spectroscopy, FTIR and scanning electron microscopy. SPE was used to preconcentrate the analytes, and the main parameters affecting the extraction, such as pH, sorbent amount, organic modifier percentage, ionic strength and elution volume, were optimized. PAEs were determined by UHPLC-PDA under gradient elution. The developed method was linear in the range 0.25–1000 ng/mL, with LOQs between 0.25 and 0.10 ng/mL and LODs between 0.008 and 0.003 ng/mL. Recovery ranged from 95.9 to 104.7%, the precision expressed as RSD% was below 7.32, and the accuracy expressed as BIAS% ranged from −5.75 to 5.93. The proposed approach provides a simple and low-cost valorization route for PPE waste, while enabling reliable PAEs analysis in drinking water.

## 1. Introduction

This historical period is often described as the “era of plastics”, characterized by the unprecedented production of polymer-based materials due to their versatility, durability, availability, and low manufacturing cost [1,2]. However, environmental persistence of plastics remains a major concern. Because of their chemical inertness, most plastics undergo extremely slow degradation, with estimates ranging between 250 and 500 years under natural environmental conditions [3,4].

Continuous industrial and commercial demand has further increased plastic production, contributing to greenhouse gas emissions and waste accumulation throughout the value chain.

If no changes in waste management systems are implemented, an estimated 12,000 Mt of plastics could accumulate in landfills and natural ecosystems by 2050 [5,6]. Currently, only 9% of plastic waste is recycled, 12% is incinerated, and the remainder is landfilled or released into the environment. Under present conditions, achieving a “zero-waste” scenario appears unrealistic; therefore, a more feasible goal consists of reducing waste generation and promoting reuse strategies aligned with the circular economy [7]. As a consequence, the implementation of circular economy strategies aimed at reducing waste generation and valorizing plastic residues is becoming increasingly relevant at the international level [8,9]. To mitigate plastic pollution, several governmental policies have been introduced, including bans on low-recyclability single-use items and the promotion of reusable alternatives such as textile-based shopping bags [10].

The global COVID-19 pandemic represented an unprecedented perturbation of the plastic waste cycle. While initial lockdown phases temporarily reduced waste generation, the massive deployment of personal protective equipment (PPE) sharply increased the production of short-lifetime polymeric materials requiring disposal [11,12]. In the post-pandemic phase, mask usage declined from its peak, yet remained common in healthcare settings, public infrastructure, and densely populated environments, sustained by an increased awareness of airborne transmission and the perceived efficacy of respiratory protection against both SARS-CoV-2 and seasonal infections. Consequently, disposable surgical masks have transitioned from emergency equipment to low-cost protective commodities, reinforcing convenience-driven single-use practices and aggravating short-cycle plastic waste streams. Recent life-cycle assessments estimated that masks used between 2020 and 2022 generated more than 18 million tons of carbon emissions [13], underscoring the environmental relevance of treating PPE as purely disposable materials. This scenario stimulated interest in recycling and valorization strategies capable of converting mask-derived polymers, predominantly polypropylene, into higher added-value materials. [14].

Surgical masks are typically composed of three layers of non-woven polypropylene (PP) with fiber diameters in the sub-micrometric to micrometric range (<1–10 µm). Polymers such as PP, polystyrene, polycarbonate, polyethylene, and polyester can be converted into non-woven sheets through spunbond or electrostatic deposition processes. [15]. Among various valorization strategies, controlled calcination under inert atmosphere has been investigated as an effective approach for producing carbon-based materials, while ensuring safe viral inactivation during processing. Carbonaceous materials obtained through polymer carbonization have been successfully applied to absorption, filtration, and adsorption systems [16,17,18]. In parallel, alternative strategies have focused on extending the operational lifetimes of surgical masks by means of sterilization procedures and physical or mechanical treatments aimed at preserving filtration performance [19,20,21].

Phthalic acid esters, derived from 1,2-benzenedicarboxylic acid, are widely used as additives in the production of plastic materials to improve the flexibility, durability, and stability of the final product [22]. Global production exceeds several million tons per year, and because phthalates are not covalently bound to polymeric matrices such as PVC or PET, they may migrate into biological and environmental compartments. Chronic exposure has been associated with carcinogenic, teratogenic, endocrine, and reproductive disorders [23,24,25], which highlights the need for the reliable monitoring of these compounds in environmental and food matrices. As a result, environmental protection agencies, including the US EPA, WHO, and the European Union, have established strict regulatory limits for phthalates in drinking and surface water since 2007 [26,27], primarily targeting industrial discharges, wastewater treatment plants, and diffuse urban pollution sources while emphasizing the critical need for reliable analytical methods to monitor these contaminants in environmental and food matrices. Sample preparation represents the most critical and time-consuming step in phthalate analysis and may introduce non-negligible systematic and random analytical errors when not properly optimized. Current approaches span adsorbent-based techniques (SPE, SPME, MSPE, MEPS, SBSE) and solvent-based procedures (LLE, DLLME, HF-LPME, DLLME-SFO) [28,29,30], typically coupled with liquid or gas chromatography for quantification [31], reinforcing the need for robust SPE workflows and efficient sorbent materials for trace-level phthalate determination. Activated carbonaceous materials have attracted increasing interest due to their highly developed porous structure, which promotes strong interactions with a wide range of chemical species, and the possibility of tuning their surface chemical composition through appropriate functionalization strategies. These characteristics allow fine control over adsorption mechanisms and selectivity, enabling the efficient removal of both organic and inorganic pollutants in environmental and biological systems [32].

Carbon-rich precursors of natural or synthetic origin can be converted into amorphous carbon, carbon fibers, carbon spheres, graphite, and nanostructured carbon through controlled thermal treatment. Although several polymers have been successfully employed as precursors for advanced carbon materials, polypropylene exhibits low thermal stability and cannot be directly carbonized at high temperature without undergoing extensive degradation. To overcome this limitation, chemical pre-treatments such as sulfonation have been investigated to enhance thermal resistance and increase the final carbon yield during calcination [33,34].

The aim of this work was to convert waste surgical masks into a carbon-based sorbent material suitable for the solid-phase extraction of phthalates from bottled water. To overcome the well-documented low thermal stability of polypropylene, we adopted a solvothermal sulfonation protocol previously shown to be effective for polyethylene and was successfully adapted to surgical mask non-woven fabric [35]. The present results demonstrate that the same approach can be effectively applied in our case.

This pre-treatment enabled sulfur incorporation into the polymer structure and promoted extensive crosslinking of the polypropylene backbone. Such structural modification was crucial for obtaining high carbon yields (~30–40%) and maintaining the fibrous morphology upon calcination, in contrast to untreated PP [36]. This upcycling approach aligns well with emerging lifecycle assessment recommendations that identify the thermal conversion of mask waste into sorbents as a promising future strategy for mitigating PPE plastic pollution [37].

The resulting sorbent was thoroughly characterized and its extraction performance systematically evaluated. Additionally, a UHPLC-PDA method was developed for phthalate quantification, achieving analytical figures of merit comparable to or exceeding those of literature methods using identical instrumentation. To the best of our knowledge, this represents the first integration of sulfonated polypropylene mask-derived carbon within a solid-phase extraction workflow specifically targeting phthalate determination in bottled water, despite growing interest in both PPE-upcycling strategies and phthalate monitoring in consumer products.

## 2. Results and Discussion

### 2.1. Characterization of the Sorbent

**Figure 1:**
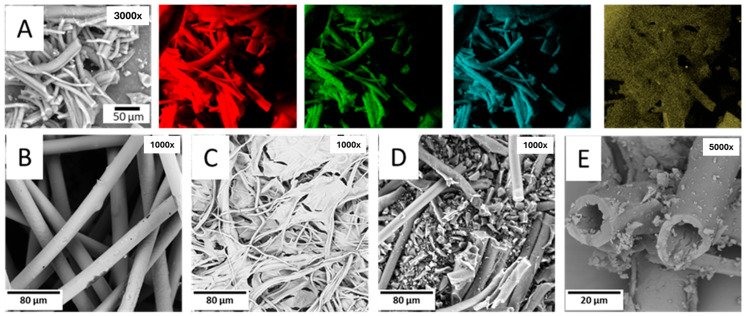
Morphological evolution during sorbent preparation. (**A**) Elemental X-ray mapping of the final product showing homogeneous distributions of C, N, O, and S (3000×). (**B**) Pristine fibers after washing and drying (1000×), (**C**) sample after sulfuric acid treatment showing modification of the surface features and partial loss of the polymeric component (1000×), (**D**) calcined and pre-ground sample by manual and mechanical grinding (1000×), and (**E**) final material (5000×).

The methodology employed for the preparation of the investigated material derived from recycled surgical masks is shortly depicted in Figure 1. SEM images revealed the heterogeneous nature of the sample. The polypropylene fibers are well distinguished, indicating that the established protocol for the preparation did not induce relevant morphological changes for the most significant portion of the sample, but rather fragmented the initial material while preserving its original fibrous structure. Figure 1 illustrates the morphology of the pristine sample and after different preparation steps. Sulfonation introduces polar sulfonic functionalities into the polypropylene backbone and promotes dehydrogenation, unsaturation, and intermolecular crosslinking reactions. These structural modifications reduce chain mobility and inhibit thermoplastic softening during thermal treatment. As a result, the fibrous morphology is preserved, and the material evolves towards a thermally stabilized structure that favors char formation rather than melt flow or volatilization. This stabilization mechanism is widely recognized in sulfonated polyolefin precursors and supports the improved carbon yield and morphology retention observed after carbonization [36]. The preservation of fibrous morphology supports the stabilization and crosslinking mechanism proposed after sulfonation.

**Figure 2:**
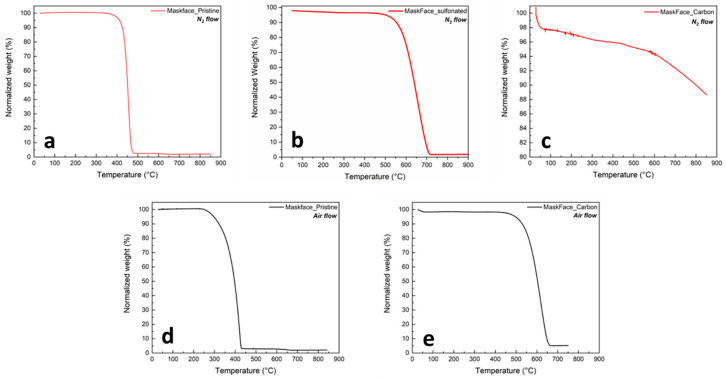
TGA curves of surgical mask-derived samples under nitrogen (**a**–**c**) and air (**d**,**e**). Under nitrogen, the pristine (**a**), sulfonated (**b**), and carbonized (**c**) samples show increasing thermal stability and residual mass, with the carbonized material retaining >85% at 900 °C. Under air, the pristine (**d**) undergoes complete oxidation between 280 and 400 °C, while the carbonized sample (**e**) decomposes above 500 °C with minor inorganic residue.

The X-ray mapping analysis of the final product identifies the constituent elements of the sample, showing that they are distributed homogenously (carbon in red, nitrogen in green, oxygen in blue, and sulfur in yellow). The pristine sample (Figure 1B) displayed several surface imperfections owing to its ‘waste nature’, while the fibers exhibited diameters twice in size with respect to the final dimension. This is evident comparing Figure 1C,D; the presence of small particles (Figure 1D), obtained from grinding the sample initially with a hand mortar and later with a ball mill, is observed, consistently remaining below or near the size of the utilized sieve. The sample retained the separated fibrous portion after the mild grinding using a mortar, however inevitably losing the entire polymer segment post sulfuric acid treatment. Figure 1E showed that the final fibers developed cavities and surface irregularities as a consequence of grinding and acid treatment. Based on thermogravimetric analyses (Figure 2), it is evident that the pristine material is thermally stable at temperatures exceeding 200 °C in both air and nitrogen. As such, the temperature set during the sulfonation did not compromise the chemical structure of the sample, confirming SEM results. Thermal analyses of the sulfuric acid-treated polypropylene subjected to calcination revealed a distinct modification in the carbon structure. Notably, the treated PP exhibited exceptional stability in both air and nitrogen, as reported in Figure 2b. A weight loss of 10% is observed for the pristine, the sulfuric acid-treated, and the final samples at temperatures of 425 °C, 552 °C, and 800 °C, respectively. The thermal stability of the pristine and final samples was also studied under oxygen-only atmosphere, in this case observing a 10% weight loss at temperatures of 322 °C and 529 °C, respectively. This behavior is likely due to structural stabilization and reduced chain mobility induced by sulfonation, which also contributes to enhanced resistance to oxidative degradation.

**Figure 3:**
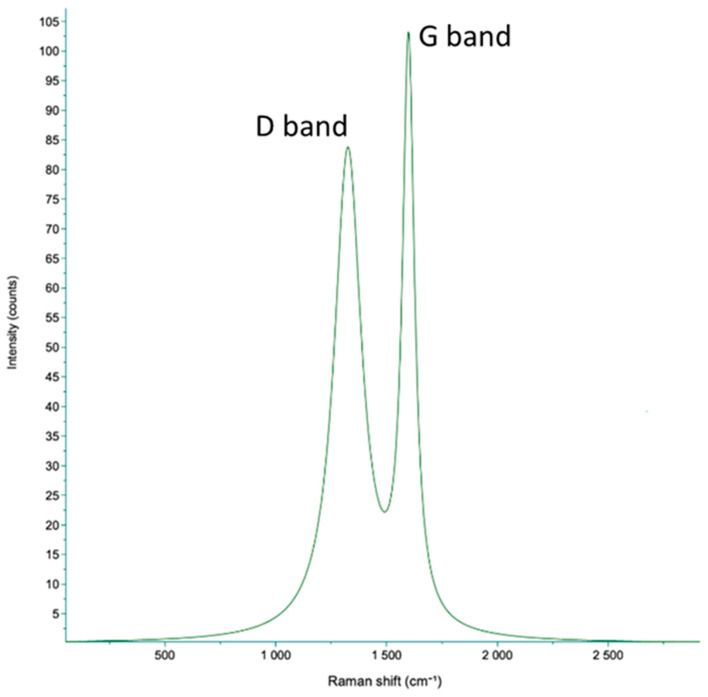
Raman spectrum of the carbonized surgical mask-derived sorbent showing the characteristic D (~1350 cm^−1^) and G (~1580 cm^−1^) bands associated with disordered and graphitic carbon domains.

Raman spectroscopy was carried out to explore the carbon nature of the obtained sample. The spectrum as shown in Figure 3 reveals two distinct characteristic peaks of carbon: the G’ band at 1597 cm^−1^, exhibiting higher intensity and narrower width, and the D’ band at 1370 cm^−1^, with lower intensity and a broader shape at its base. The spectrum obtained suggests the presence of a considerable amount of amorphous material in the sample. This observation aligns with the calcination temperature utilized during the preparation, which did not exceed 650 °C [38].

**Figure 4:**
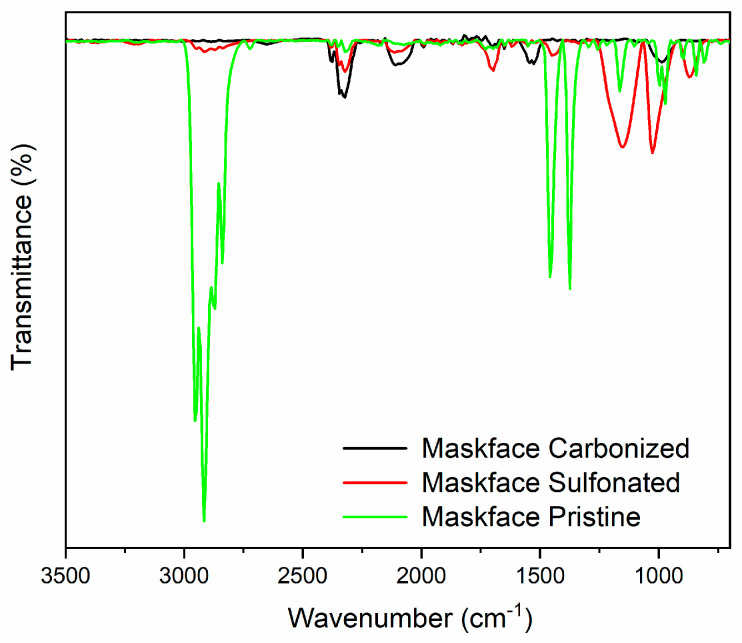
FTIR spectra of surgical mask samples.

The FTIR spectra showing the characteristic bands of the pristine, sulfonated, and final samples are compared in Figure 4 [34]. The spectrum of the pristine sample adheres to the typical characteristics of PP, and, at a compositional level, the main constituent groups are indeed distinguishable. In particular, the presence of methyl groups is observable through the bands around 1000 cm^−1^ related to rocking vibrations and around 1350 and 2970 cm^−1^ with the symmetric bending and asymmetric stretching vibrations, respectively. Additionally, the peaks at 1450, 2850, and 2900 cm^−1^ are attributed to symmetric bending, symmetric stretching, and asymmetric stretching of the -CH_2_- groups, respectively. After the initial sulfonation the characteristic peaks associated with the PP phase disappeared. In the sulfonated samples, the formation of double bonds and sulfonic acid groups takes place along the PP structure, resulting in the disappearance or decreased intensity of the peaks associated with -CH, -CH_2_, and -CH_3_ groups. In the red spectrum, distinct peaks in the range from 1250 to 1000 cm^−1^, linked to the presence of sulfonic acid groups, are evident. Peaks around 1700 and 1600 cm^−1^ indicate the presence of bonds attributable to carbonyl groups and -C=C- bonds within the sample’s structure. Regarding the final sample, the presence of sulfur-linked groups is confirmed through very weak stretching values of thiol groups, although they are less prominent than those generated in the sulfonation phase. Bands related to bonds assignable to carbonyl groups and -C=C- bonds in the sample’s structure are still distinguishable. These spectral changes support the chemical modification induced by sulfonation and are consistent with the stabilization mechanism described above.

### 2.2. Optimization of the Extraction Conditions

The investigated PAEs were used as model analytes to evaluate the solid-phase extraction performance of the prepared sorbent. Several parameters, including the sample pH, the amount of sorbent, the methanol content in sample loading, the ionic strength, the elution solvent, and its volume were considered and optimized. All the experiments were conducted using standard solutions of PAEs at the concentration of 1000 ng/mL in triplicate.

#### 2.2.1. Effect of the Amount of Sorbent

**Figure 5:**
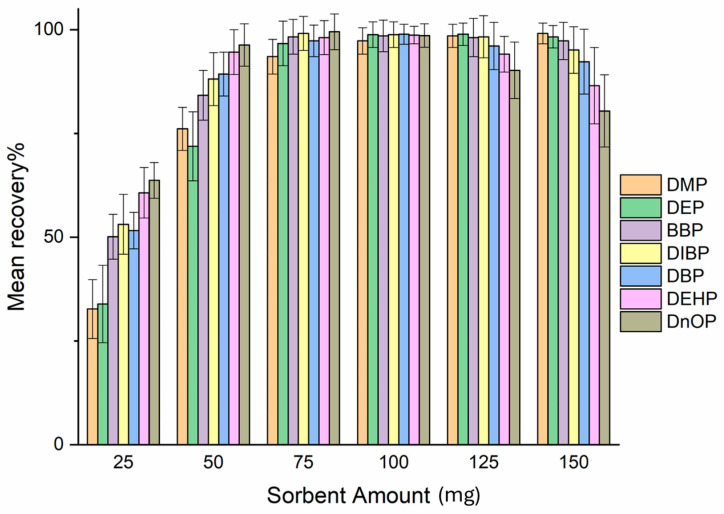
Effect of the amount of sorbent on the recovery of PAEs.

Numerous experiments were performed to assess the quantity of sorbent for the complete adsorption of the investigated PAEs. Several emptied SPE cartridges were packed with 25–50–75–100–125–150 mg of sorbent and loaded with PAEs. As an effective adsorption control, the eluate obtained in the loading phase was then analyzed in UHPLC to verify the possible presence of non-adsorbed PAEs. As shown in Figure 5, 75 mg of sorbent is necessary for the complete adsorption of the analytes. For amounts greater than 100 mg, it has been seen that the eluting solvent was unable to completely elute the cartridge with a consequent decrease in recovery. For these reasons, 100 mg of sorbent was chosen as the optimal quantity, and the consequent experiments were carried out using this amount of sorbent.

#### 2.2.2. Effect of pH

**Figure 6:**
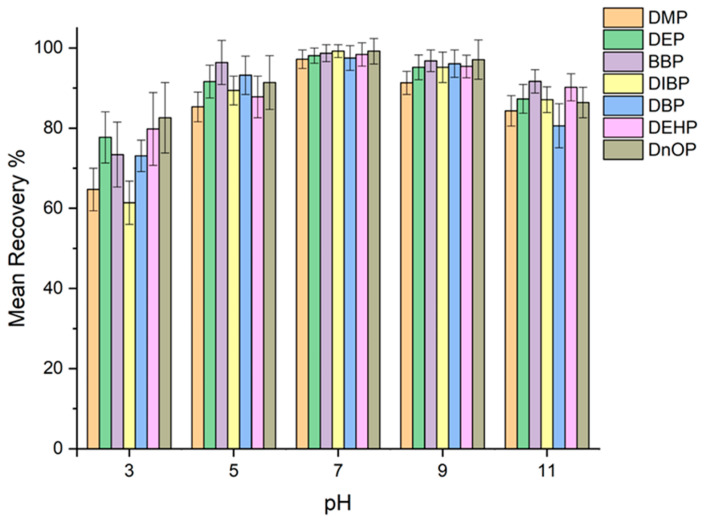
Effect of sample pH on recovery in the SPE procedure.

Loading pH is a key parameter in the adsorption of an analyte by a sorbent. Although PAEs do not possess ionizable groups in their structure that would allow the non-dissociated form to be more readily retained through a reversed-phase retention mechanism, being dominated by hydrophobic interactions and π–π interactions, different pH values ranging from 3 to 11 were evaluated using acetate and phosphate buffer. It has been seen, as shown in Figure 6, that the phosphate buffer at pH 7 has the best performance in terms of adsorption of PAEs. This phenomenon can be explained by the possible hydrogen bonds that are established between the sorbent and the analytes at that pH; at pH ranging between 3 and 6, there is a decrease in the adsorption of PAEs as the acidic pH causes this type of interaction to decrease. Any potential formation of hydrogen bonds at this specific pH value would add to the already existing hydrophobic interactions, resulting in a slight increase in the retention of the analytes within the SPE cartridge. At pH greater than 7, a small decrease in recovery was noted. This data can be explained because, as reported in the literature, PAEs at basic pH can undergo hydrolysis by decreasing their adsorption. For this reason, pH 7 was chosen for further experiments.

#### 2.2.3. Effect of Organic Modifier

Since phthalates, especially long-chain ones, have a great affinity for plastic materials such as SPE cartridges, it is good practice to add a small percentage of an organic modifier to avoid adsorption of PAEs along the walls of the cartridge. In this study, different percentages of acetonitrile between 0 and 15% were added to evaluate the interference between the SPE cartridge and phthalates.

**Figure 7:**
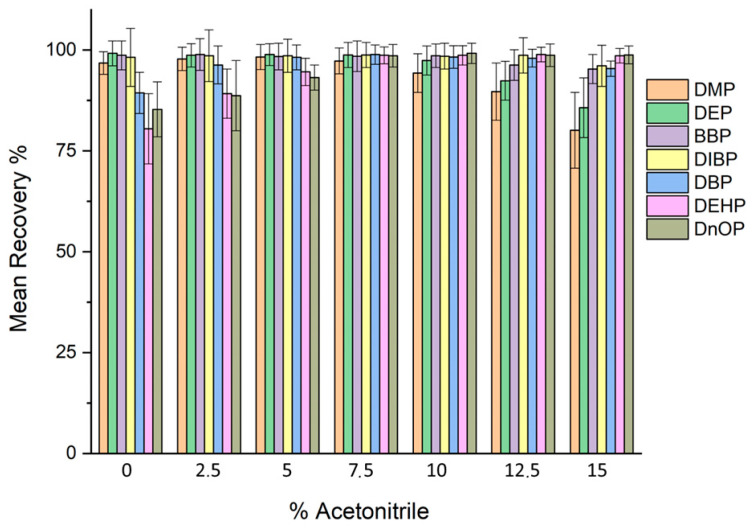
Effect of organic modifier on recovery in the SPE procedure.

As shown in Figure 7, there was an increase in the recovery of DBP, DEHP, and DnOP passing from 0% to 7.5%, while no improvement was observed in the recovery of the other PAEs. For higher percentages of acetonitrile, on the other hand, it was seen that the recovery of DMP, DEP, BBP, and DIBP worsened while the recovery of DBP, DEHP, and DnOP remained constant. For this reason, a percentage of organic modifier, equal to 7.5% *v*/*v*, was chosen.

#### 2.2.4. Effect of Ionic Strength

The ionic strength was evaluated at different concentrations of sodium chloride. Usually, the addition of salt, on the one hand, decreases the solubility of an organic compound in water; on the other hand, it increases the viscosity of the solution, which is going to decrease the distribution coefficient. In this study, it was seen that 5% NaCl (*w*/*v*) can increase the adsorption of shorter chain phthalates such as DMP and DEP while having a negligible effect on all other phthalates. However, higher concentrations of NaCl resulted in decreased recovery of phthalates, which was why 5% *w*/*v* sodium chloride was added to the samples in subsequent experiments.

#### 2.2.5. Effect of the Elution Solvents and Its Volume

Elution is a key process in solid-phase extraction, because an unsuitable solvent could not desorb totally the PAEs negatively affecting the recovery. Different solvents were tested in this study, including methanol, acetonitrile, acetone, and isopropanol, alone or in combination. The use of acetonitrile, methanol, and acetone promote the elution of DMP, DEP, and DnOP, while the use of isopropanol increases the elution of BBP, DBP, DIBP, and DEHP. Using a mixture composed of 50% *v*/*v* of acetonitrile and isopropanol, a complete elution of all the analytes was obtained. To determine the smallest volume that could give the complete elution of all the analytes, different volumes of this mixture were also tested, with the result that 1 mL of acetonitrile and isopropanol 50/50 *v*/*v* was sufficient for the complete elution of PAEs.

### 2.3. Evaluation of Breakthrough Volume

To increase the sensitivity of the method, it is necessary to obtain a high enrichment factor which can be obtained by increasing the volumes of the sample percolated in the SPE cartridge. However, increasing sample volume can lead to an unexpected loss of analytes during the enrichment process. To fully understand the mechanisms underlying SPE and increase their efficiency, it is essential to know the parameters that influence it, such as breakthrough volume (Vb), retention volume (Vr), hold-up volume (Vm), retention factor (k), and theoretical plates number (N). In this work, to determine the breakthrough volume, a frontal analysis was conducted using solutions containing the analytes at a concentration of 50 µg/mL and continuously percolated into a previously packed and conditioned SPE cartridge [39]. Under ideal conditions, the experimental curve (concentration vs volume of solution) has a bilogarithmic shape and, once fitted using the Boltzmann’s equation, the regression parameters are used to obtain the values of Vb, Vr, Vm, k, and N. Table 1 shows the parameters relating to the prepared sorbent, obtained using the mathematical approach reported in the literature [39]. Analyzing these parameters, it is evident how the sorbent has a remarkable affinity for the tested phthalates.

### 2.4. Method Evaluation and Sample Analysis

The developed method was subsequently validated in accordance with the guidelines [40]. For this purpose, the selectivity was evaluated using six different sources of water bottled in glass containers and subjected to the proposed method. No signal attributable to interferents was observed at the retention time of the studied PAEs. A series of experiments were subsequently performed to determine the limits of detection (LODs) and the limits of quantification (LOQs) of the investigated PAEs. The calibration curves were linear in the range of 0.25–1000 ng/mL, the LOQs of the PAEs analyzed were between 0.25 and 0.10 ng/mL, while the LODs were between 0.008 and 0.003 ng/mL. The precision and accuracy were calculated using the quality control samples obtained, starting from stock solutions prepared independently from the stock solutions used to obtain the calibration curve. The results are reported in Table 2. The enrichment factor (EF) was calculated as the ratio between the concentration of phthalates in 100 µL of isopropanol and the initial concentration in the aqueous sample, showing EF ranging between 98 and 156, demonstrating good efficiency in the extraction and pre-concentration of the samples containing the phthalates.

Finally, the performance of the sorbent proposed in this study was tested in plastic bottled water samples. To check the extractive yield, the same samples were analyzed twice, once without adding PAEs, and the second time adding a standard solution 25 ng/mL of PAEs. The results in Table 3 demonstrate that phthalates with the longest chains are detected the most. The recoveries obtained after the addition of the phthalates were in the range of 95.9% and 104.7%.

**Figure 8:**
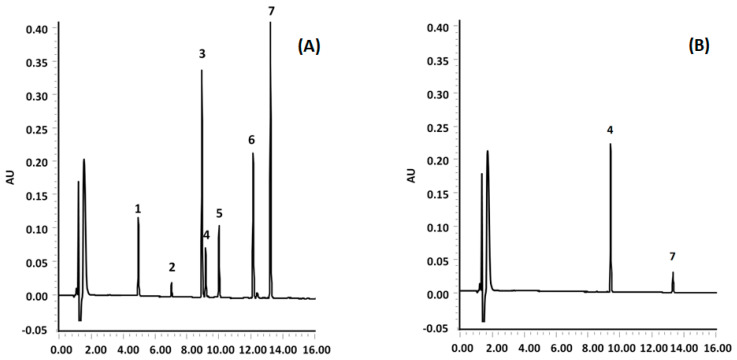
UHPLC-PDA chromatograms of a spiked blank sample added with standard solution of the investigated PAEs (**A**), and a sample subjected to the proposed method (**B**), respectively. Peak identification: 1 DMP, 2 DEP, 3 BBP, 4 DiBP, 5 DBP, 6 DEHP, 7 DnOP.

This data suggests that the produced sorbent can be used in the SPE of phthalates with different polarities, maintaining excellent performance as shown in the chromatograms reported in Figure 8.

### 2.5. Comparison with Methods Reported in the Literature

The performance of the developed method was compared with some others’ in the literature data. Many works reported the simultaneous determination of five or more phthalates using different sample preparation techniques that can be divided between those deriving from liquid–liquid extraction (LPME and DLLME) and those making use of sorbents (SBME, SPME, SPE). Looking at the data collected in Table 4, the method proposed here is comparable or even superior to some already existing methods. Furthermore, the sorbent was prepared by giving added value to waste such as surgical masks, for which disposal is a serious problem [28,41,42,43,44,45,46,47,48,49,50,51].

## 3. Experimental

### 3.1. Reagents and Chemicals

Dimethyl phthalate (DMP), diethyl phthalate (DEP), di-isobutyl-phthalate (DiBP), dibutyl phthalate (DBP), benzyl butyl phthalate (BBP), di-2-ethylhexyl phthalate (DEHP), n-octyl-phthalate (DnOP), sodium chloride, and sodium hydroxide were purchased from Sigma-Aldrich (Milan, Italy). Acetonitrile, methanol, 2-propanol, formic acid, and 96 wt % sulfuric acid were supplied by Carlo Erba Reagenti (Milan, Italy). HPLC grade water was prepared by passing water through an Elix 3 and a Milli-Q Academic water purification system. Polypropylene-based surgical masks were sourced from post-consumer, non-clinical environments.

### 3.2. Apparatus

The chromatographic separation was obtained using a UHPLC (H-Class Waters) equipped with a quaternary pump, an autosampler, a column heater and a degassing system. The determination of phthalates was performed using a Waters 2996 diode array detector (Waters, Milford, MA, USA). The column used was a Kinetex XB C18 (Phenomenex, Torrance, CA, USA, 75 mm, 2.1 ID, 1.7 µm particle size). The mobile phase used was a mixture of 0.1% formic acid in water (line A) and 0.1% formic acid in acetonitrile-isopropyl alcohol (75-25 *v*/*v*, line B) in gradient elution. The percentage of line B used in the gradient elution program was as follows: 0–8 min 50–70%B, 8–10 min 70–99%B, 10–14 99%B, 14–16 45%B. The flow rate was 0.75 mL min^−1^ and the injection volume was 5 µL. An analytical balance “Precisa” model XT120A was used while a Labsonic ultrasonic bath (FALC, Milan, Italy) was used for sonication and an Eppendorf Centrifugate 5804 was used to centrifuge the samples.

### 3.3. Preparation of Working Solutions

Stock solutions were individually prepared by withdrawing an exact volume of standard solution with a micropipette and diluting it with acetonitrile in a 10 mL volumetric flask to obtain a stock solution at a concentration of 1 mg/mL. The PAEs stock solutions were prepared by mixing the stock solutions of all the investigated PAEs and diluting them with acetonitrile up to a concentration of 100 µg/mL. Stock solutions were placed in glass vials, protected from light, and stored at −20 °C. To avoid any possible contamination, all glassware were washed with methanol three times. Working standard solutions at different concentrations (0.1 to 1000 ng/mL) were prepared weekly by diluting the PAEs stock solution with a solution consisting of Milli-Q water and acetonitrile (75/25 *v*/*v*). All the working solutions were stored at 4 °C.

### 3.4. Preparation of Sorbent

**Figure 9:**
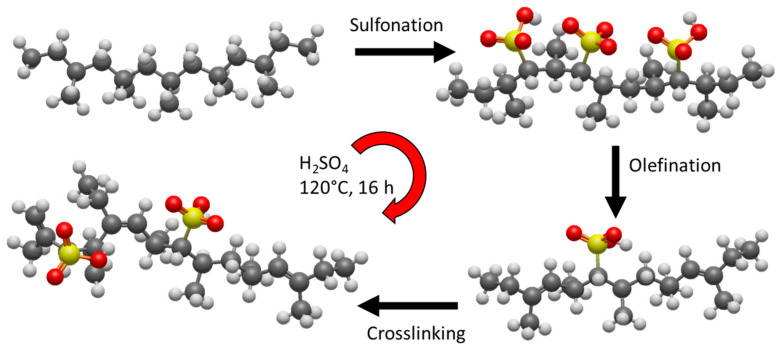
Schematic representation of the preparation of the polypropylene-based sorbent via sulfonation, olefination, and crosslinking at 120 °C.

The surgical masks were washed five times with water and methanol to remove dirty and possible contaminants and then left in the oven at 90 °C for 24 h. The elastic and the metal nose piece were removed, and the rest was cut into uniform strips and immersed in a hermetic Teflon container containing sulfuric acid, then transferred to an oven (Carbolite GERO, HOPE, Kettering, UK) and left at 120 °C for about 16 h. At this point, the solution was neutralized with sodium hydroxide, and the material was washed with Milli-q Water until pH neutrality. The sample thus obtained was placed in a quartz cell and heated under a nitrogen flow of 50 cc/min with a ramp of 5 °C/min up to 650 °C with a final isotherm of 2 h. The material thus obtained was ground for about 2 h using ZrO_2_ beads in a PM100 planetary ball mill (Retsch GmbH, Haan, Germany) and then sieved to make the sample as homogeneous as possible, using sieves with woven wire mesh and perforated plates (Controls, Milan, Italy), up to a size of 0.075 mm. Figure 9 shows the chemical mechanism and the key stages involved in the sample preparation. It began with the initial sulfonation of polypropylene, followed by the olefination reaction, and subsequent processes of addition and rearrangement. At the end, the PP chains underwent a crosslinking process. Throughout the protocol, a constant temperature of 120 °C was maintained to avoid any decomposition.

### 3.5. Characterization of the Sorbent

Morphology was studied by acquiring Scanning Electron Microscopy (SEM) images using a Phenom XL Desktop apparatus (Alfatest, Milan, Italy) in high vacuum with an accelerating voltage of 15 KV and an optical magnification of 3000× for sample A, 1000× for samples B, C, and D, and 5000× for sample E. Phenom ProSuite software was used for size measurement, elemental analysis, and atomic distribution. Fourier transform infrared (FTIR) spectra were acquired using a Shimadzu IRAffinity-1S FTIR (Shimadzu Italia Srl, Milan, Italy) spectrophotometer. The software used for the spectra manipulation was LabSolution IR version 2.27. A Horiba XploRA™ PLUS Raman microscope (Horiba Italia Srl, Roma, Italy) was used to obtain Raman spectroscopy analyses using a 50×_VIS objective and 532 nm edge laser and the LabSpec 6.6.1.14 spectroscopy suite software. Thermogravimetric analyses were obtained through a PerkinElmer STA6000 (Perten Instruments Italia Srl, Milan, Italy) with Pyris software Version 11. In addition to the final samples, the pristine ones were also processed, and each was heated both in air, with a flow of 20 mL/min at a rate of 10 °C/min at 850 °C, and in nitrogen with a flow of 20 mL /min at a rate of 10 °C/min at 850 °C.

### 3.6. SPE Preconcentration Procedure

Solid-phase extraction of the investigated PAEs was performed on a Visiprep 12 solid Phase Extraction Vacuum Manifold using a vacuum (Sigma Aldrich, St. Louis, MO, USA). A 1 mL SPE cartridge previously emptied was packed with 100 mg of the prepared sorbent. The sorbent was conditioned with 3 × 1 mL of acetonitrile and subsequently equilibrated with 3 × 1 mL of Milli-Q water. Then, 50 mg of sodium chloride and 750 µL of acetonitrile were added to 10 mL of water from plastic bottles, and were then loaded into the cartridge 1 mL at a time. The cartridge was subsequently washed using 2 × 1 mL of Milli-Q water and finally eluted using 1 mL of a mixture of acetonitrile and isopropyl alcohol (50/50 *v*/*v*). The resulting solution was dried under nitrogen steam and reconstituted with 100 µL of isopropanol. The eluate was then vortexed for 1 min, filtered on Phenex-PTFE (4 mm, 0.45 µm) filters (Phenomenex, Torrance, CA, USA), and 5 µL was injected into the UHPLC system.

## 4. Conclusions

In conclusion, in this work, a sorbent was prepared starting from waste surgical masks, to be used in the solid-phase extraction of Phthalic acid esters in bottled water samples and their determination by UHPLC-PDA. The sorbent was derived from waste materials through chemical and thermal treatments, resulting in enhanced thermal stability. Using a set of optimized conditions, the prepared sorbent obtained good performances in terms of recovery and enrichment which are comparable or superior to those reported in the literature. The sorbent developed in this study is not limited to phthalate removal, but is potentially applicable to a broader range of pollutants in environmental and biological matrices. This study contributes to waste-valorization strategies within a circular economy framework by proposing a simple and potentially scalable waste-to-resource pathway consistent with green chemistry principles. The use of low-cost waste precursors and operationally straightforward processing highlights its practical feasibility and its potential for sustainable implementation. Comprehensive environmental and techno-economic assessments will be essential to further substantiate its viability at an industrial scale. Overall, this work provides a straightforward example of waste-to-resource conversion, in which scientific knowledge supports the transformation of discarded materials into value-added sorbents for environmental applications.

## Figures and Tables

**Table 1:** Parameters obtained from frontal analyses of PAEs solutions (50 µg/mL) using the prepared sorbent.

Analyte	Breakthrough Volume, V_B_ (mL)	Retention Volume, V_R_ (mL)	Equilibrium Volume, V_E_ (mL)	Retention Factor, K	Theoretical Plates, N
**DMP**	11.75	29.63	33.50	0.854	109
**DEP**	15.38	38.75	43.71	0.863	109
**BBP**	28.52	60.65	65.81	0.906	137
**DiBP**	28.64	62.03	67.39	0.905	140
**DBP**	29.09	62.93	68.48	0.904	143
**DEHP**	29.86	64.72	68.88	0.925	144
**DnOP**	32.49	72.54	77.60	0.921	148

**Table 2:** Inter-day and intra-day precision and accuracy of the proposed method.

Analyte	Amount Added (ng/mL)	Intra-Day		Inter-Day	
		Accuracy (BIAS%)	Precision (RSD%)	Accuracy (BIAS%)	Precision (RSD%)
**DMP**	0.25	2.88	2.27	4.36	3.07
	0.75	−3.76	1.58	−5.38	2.20
	25	3.52	3.52	5.11	4.07
	850	4.22	4.89	5.93	5.21
**DEP**	0.25	3.30	3.68	4.84	6.32
	0.75	−2.53	1.60	−3.95	1.46
	25	2.65	1.83	4.10	1.65
	850	2.85	0.19	4.32	0.28
**BBP**	0.25	3.38	3.25	4.95	2.84
	0.75	2.70	1.93	4.15	1.73
	25	2.43	1.01	3.83	0.96
	850	−2.35	1.16	−3.74	1.09
**DIBP**	0.25	3.27	3.02	4.81	2.65
	0.75	3.43	0.94	5.00	2.67
	25	−4.07	2.19	−5.75	1.71
	850	2.58	3.21	4.01	6.78
**DBP**	0.25	3.30	4.42	4.84	7.32
	0.75	2.53	1.60	3.95	1.46
	25	2.65	1.83	4.10	1.65
	850	2.85	0.19	4.32	1.28
**DEHP**	0.25	3.38	3.25	4.95	2.84
	0.75	−2.70	1.93	−4.15	1.73
	25	2.43	1.01	3.83	0.96
	850	2.35	1.16	3.74	1.09
**DnOP**	0.25	3.27	3.02	4.81	2.65
	0.75	3.43	0.94	5.00	0.67
	25	4.07	2.19	5.75	5.26
	850	2.58	0.71	4.01	0.71

**Table 3:** Recovery and results of the proposed SPE–UHPLC-PDA method.

SAMPLES	ANALYTES							
		*DMP*	*DEP*	*BBP*	*DiBP*	*DBP*	*DEHP*	*DnOP*
**SAMPLE #1**	*Added* (ng/mL)	0	0	0	0	0	0	0
	*Determined*	Nd	0.94	2.80	Nd	Nd	3.60	Nd
	*Added* (ng/mL)	25.00	25.00	25.00	25.00	25.00	25.00	25.00
	*Determined*	24.06	25.91	28.13	25.00	24.87	28.47	25.09
**SAMPLE #2**	*Added* (ng/mL)	0	0	0	0	0	0	0
	*Determined*	Nd	Nd	Nd	Nd	Nd	Nd	Nd
	*Added* (ng/mL)	25.00	25.00	25.00	25.00	25.00	25.00	25.00
	*Determined*	25.12	25.01	25.00	24.76	25.08	24.81	23.98
**SAMPLE #3**	*Added* (ng/mL)	0	0	0	0	0	0	0
	*Determined*	Nd	Nd	Nd	8.71	Nd	Nd	0.8
	*Added* (ng/mL)	25.00	25.00	25.00	25.00	25.00	25.00	25.00
	*Determined*	25.85	24.85	24.12	32.4	25.09	24.21	25.88
**SAMPLE #4**	*Added* (ng/mL)	0	0	0	0	0	0	0
	*Determined*	Nd	1.8	0.66	Nd	Nd	Nd	1.32
	*Added* (ng/mL)	25.00	25.00	25.00	25.00	25.00	25.00	25.00
	*Determined*	24.6	26.5	25.55	25.1	25.00	24.21	26.12
**SAMPLE #5**	*Added* (ng/mL)	0	0	0	0	0	0	0
	*Determined*	Nd	Nd	Nd	Nd	2.13	Nd	1.97
	*Added* (ng/mL)	25.00	25.00	25.00	25.00	25.00	25.00	25.00
	*Determined*	25.04	24.75	25.40	24.67	26.76	25.10	26.12
**SAMPLE #6**	*Added* (ng/mL)	0	0	0	0	0	0	0
	*Determined*	2.4	Nd	2.30	Nd	Nd	6.13	Nd
	*Added* (ng/mL)	25.00	25.00	25.00	25.00	25.00	25.00	25.00
	*Determined*	27.51	25.40	27.51	24.87	25.41	32.6	24.53

Nd: not detected (˂LOQ).

**Table 4:** Comparison of the methods reported in the literature with the proposed method.

Sorbent	Method	LOD (ng/mL)	LOQ (ng/mL)	Recoveries (%)	Sample	Ref.
Fe_3_O_4_@PDA	GC-MS	-	0.5	71–120	Mineral water, wastewater	[41]
MWCNTs/GO	GC-FID	0.036–1.41	1.0	86.4–103.5	Seawater	[28]
PDA/MF aerogel	HPLC-PDA	1.2–2.2	5–10	80–116	Surface water, bottled water	[42]
[C12 mim]Br-silica	HPLC-UV	0.12–0.17	0.5	85–107	Wastewater	[43]
[C8 mim][PF6]	HPLC-UV	0.68–1.36	-	91.4–94.3	River water	[44]
Polychloroprene	HPLC-UV	1.2–2.2	5–50	82.7–116.9	Landfill leachates	[45]
Fe_3_O_4_@G@PDA	GC-MS	0.05–1.5	50	91.4–98.7	Human plasma	[46]
Fe_3_O_4_/ZIF-67	GC-MS	0.0035–0.005	1	81.2–107.2	Lake water, drinking water	[47]
Activated Carbon	UHPLC-UV	0.18–2.95	0.72–9.83	78.8–104.6	Bottled water	[48]
MWCNTs-PVA-cryogel	GC-MS	2.6–3.6	25.0	70.0–118.0	Packaged food	[49]
MWCNTs-Fe_3_O_4_/Ag	GC-MS	0.11–0.23	0.5	99.6–109	Carbonated soft drinks	[50]
Brij 35 surfactant	HPLC-UV	0.03–0.012	0.05–0.1	88.4–98	Environmental water samples	[51]
Activated carbon-like	UHPLC-PDA	0.003–0.008	0.1–0.25	95.9–104.7	Bottled water	This work

## Data Availability

The datasets supporting the findings of this study, including the source data for all figures, are provided within the paper.

## References

[1] Shams M., Alam I., Mahbub S. (2021). Plastic pollution during COVID-19: Plastic waste directives and its long-term impact on the environment. Environ. Adv..

[2] Anagnosti L., Varvaresou A., Pavlou P., Protopapa E., Carayanni V. (2021). Worldwide actions against plastic pollution from microbeads and microplastics in cosmetics focusing on European policies. Has the issue been handled effectively?. Mar. Pollut. Bull..

[3] Chamas A., Moon H., Zheng J., Qiu Y., Tabassum T., Jang J.H., Abu-Omar M., Scott S.L., Suh S. (2020). Degradation Rates of Plastics in the Environment. ACS Sustain. Chem. Eng..

[4] Matjašič T., Simčič T., Medvešček N., Bajt O., Dreo T., Mori N. (2021). Critical evaluation of biodegradation studies on synthetic plastics through a systematic literature review. Sci. Total Environ..

[5] Silva A.L.P., Prata J.C., Walker T.R., Campos D., Duarte A.C., Soares A.M.V.M., Barcelò D., Rocha-Santos T. (2020). Rethinking and optimising plastic waste management under COVID-19 pandemic: Policy solutions based on redesign and reduction of single-use plastics and personal protective equipment. Sci. Total Environ..

[6] Boyle K., Örmeci B. (2020). Microplastics and Nanoplastics in the Freshwater and Terrestrial Environment: A Review. Water.

[7] Joanna V., Hardesty B.D. (2017). Plastic pollution challenges in marine and coastal environments: From local to global governance. Restor. Ecol..

[8] Jebaranjitham J.N., Christyraj J.D.S., Prasannan A., Rajagopalan K., Chelladurai K.S., Gnanaraja J.K.J.S. (2022). Current scenario of solid waste management techniques and challenges in COVID-19—A review. Heliyon.

[9] Ganesapillai M., Mondal B., Sarkar I., Sinha A., Ray S.S., Kwon Y.-N., Nakamura K., Govardhan K. (2022). The face behind the COVID-19 mask—A comprehensive review. Environ. Technol. Innov..

[10] Tejaswini M.S.S.R., Pathak P., Ramkrishna S., Ganesh P.S. (2022). A comprehensive review on integrative approach for sustainable management of plastic waste and its associated externalities. Sci. Total Environ..

[11] Wang C., Pan R., Wan X., Tan Y., Xu L., Ho C.S., Ho R.C. (2020). Immediate psychological responses and associated factors during the initial stage of the 2019 coronavirus disease (COVID-19) epidemic among the general population in China. Int. J. Environ. Res. Public Health.

[12] Lyu L., Bagchi M., Markoglou N., An C., Peng H., Bi H., Yang X., Sun H. (2024). Towards environmentally sustainable management: A review on the generation, degradation, and recycling of polypropylene face mask waste. J. Hazard. Mater..

[13] Li Y., Tang Y., Liu M., Yuan X., Zuo J., Feng K., Wang Q., Ma Q., Mu R., Wang W. (2023). Life-cycle assessment reveals disposable surgical masks in 2020–2022 led to more than 18 million tons of carbon emissions. One Earth.

[14] Min J.K., Zhang S., Li J.X., Klingeler R., Wen X., Chen X.C., Zhao X., Tang T., Mijowska E. (2019). From polystyrene waste to porous carbon flake and potential application in supercapacitor. Waste Manag..

[15] Chellamani K.P., Veerasubramanian D., Balaji R.V. (2013). Surgical face masks: Manufacturing methods and classification. J. Acad. Ind. Res..

[16] Bruni P., Avino P., Ferrone V., Pilato S., Barbacane N., Canale V., Carlucci G., Ferrari S. (2023). Preparation of Fe_3_O_4_-reduced graphene-activated carbon from wastepaper in the dispersive solid-phase extraction and UHPLC-PDA determination of antibiotics in human plasma. Separations.

[17] Robertson M., Obando A.G., Emery J., Qiang Z. (2022). Multifunctional Carbon Fibers from Chemical Upcycling of Mask Waste. ACS Omega.

[18] Ferrone V., Bruni P., Canale V., Sbrascini L., Nobili F., Carlucci G., Ferrari S. (2022). Simple synthesis of Fe_3_O_4_@-activated carbon from wastepaper for dispersive magnetic solid-phase extraction of non-steroidal anti-inflammatory drugs and their UHPLC–PDA determination in human plasma. Fibers.

[19] Rubio-Romero J.C., Pardo-Ferreira M., Torrecilla-García J.A., Calero-Castro S. (2020). Disposable masks: Disinfection and sterilization for reuse, and non-certified manufacturing, in the face of shortages during the COVID-19 pandemic. Saf. Sci..

[20] Pirker L., Krajnc A.P., Malec J., Radulović V., Gradišek A., Jelen A., Remškar M., Mekjavić I.B., Kovač J., Mozetič M. (2021). Sterilization of polypropylene membranes of facepiece respirators by ionizing radiation. J. Membr. Sci..

[21] Battegazzore D., Cravero F., Frache A. (2020). Is it Possible to Mechanical Recycle the Materials of the Disposable Filtering Masks?. Polymers.

[22] Carlstedt F., Jönsson B.A., Bornehag C.G. (2012). PVC flooring is related to human uptake of phthalates in infants. Indoor Air.

[23] Schwarzenbach R.P., Egli T., Hofstetter T.B., Von Gunten U., Wehrli B. (2010). Global Water Pollution and Hu-man Health. Annu. Rev. Environ. Resour..

[24] Main K.M., Mortensen G.K., Kaleva M.M., Boisen K.A., Daamgard I.N., Chellakooty M., Schmidt I.M., Suomi A., Virtanen H.E., Petersen D.V.H. (2006). Human breast milk contamination with phthalates and alteration of endogenous reproductive hormones in infants three months of age. Envirion. Health Perspect..

[25] Ventrice P., Ventrice D., Russo E., De Sarro G. (2013). Phthlates: European regulation, chemistry, pharmacokinetic and related toxicity. Environ. Toxicol. Pharmacol..

[26] Giuliani A., Zuccarini M., Cichelli A., Khan H., Reale M. (2020). Critical Review on the presence of phthalates in food and evidence of their biological impact. Int. J. Environ. Res. Public Health.

[27] Net S., Sempere R., Delmont A., Paluselli A., Ouddane B. (2015). Occurence, fate, behaviour and ecotoxicological state of phthalates in different environmental matrices. Environ. Sci. Technol..

[28] Habibi E., Ghanemi K., Larki A. (2017). Efficient extraction of phthalate ester with different polarities from seawater samples using multi walled carbon nanotubes/graphene oxide nanosheets. Anal. Methods.

[29] Ghorbani M., Aghamohammadhassan M., Ghorbani H., Zabihi A. (2020). Trends in sorbent development for dispersive micro-solid phase extraction. Microchem. J..

[30] Mohebbi M., Heydari R., Ramezani M. (2017). Solvent-vapor assisted liquid-liquid microextraction: A novel method for the determination of phthalate esters in aqueous samples using GC-MS. J. Sep. Sci..

[31] Pawliszyn J., Lord H.L. (2010). Handbook of Sample Preparation.

[32] Isaeva V.I., Vedenyapina M.D., Kurmysheva A.Y., Weichgrebe D., Nair R.R., Nguyen N.P.T., Kustov L.M. (2021). Modern carbon-based materials for adsorptive removal of organic and inorganic pollutants from water and wastewater. Molecules.

[33] Ahmed A., Verma S., Mahajan P., Sundramoorthy A.K., Arya S. (2023). Upcycling of surgical facemasks into carbon-based thin film electrode for supercapacitor technology. Sci. Rep..

[34] Younker J.M., Saito T., Hunt M.A., Naskar A.K., Beste A. (2013). Pyrolysis Pathways of Sulfonated Polyethylene, an Alternative Carbon Fiber Precursor. J. Am. Chem. Soc..

[35] Lee G., Lee M.E., Kim S.-S., Joh H.-I., Lee S. (2022). Efficient Upcycling of Polypropylene-Based Waste Disposable Masks into Hard Carbons for Anodes in Sodium Ion Batteries. J. Ind. Eng. Chem..

[36] Zhang S., Sun N., Jiang M., Soomro R.A., Xu B. (2023). Trash to treasure: Sulfonation-assisted transformation of waste masks into high-performance carbon anode for sodium-ion batteries. Carbon.

[37] UNEP (2022). Single-Use Face Masks and Their Alternatives: Environmental Impacts and Supply Chain Considerations. https://www.lifecycleinitiative.org/library/single-use-face-masks-and-their-alternatives/.

[38] Schuepfer D., Badaczewski F., Guerra-Castro J.M., Hofmann D.M., Heiliger C., Smarsly M., Klar P.J. (2020). Assessing the structural properties of graphitic and non-graphitic carbons by Raman spectroscopy. Carbon.

[39] Bielicka-Daszkiewicz K., Voelkel A. (2009). Theoretical and experimental method for determination of the breakthrough volume of SPE sorbents. Talanta.

[40] FDA Food and Drug Administration of the United States (2003). Guidance for Industry-Bioanalytical Method Validation.

[41] Gonzalez-Salamo J., Socas-Rodriguez B., Hernandez-Borges J., Rodriguez-Delgado M.A. (2017). Determination of phthalic acid esters in water samples using core-shell poly(dopamine) magnetic nanoparticles and gas chromatography tandem mass spectrometry. J. Chromatogr. A.

[42] Wang X., Feng J., Tian Y., Li C., Ji X., Luo C., Sun M. (2019). Melamine-formaldehyde functionalized with polydopamine as in-tube solid-phase microextraction coating for the determination of phthalate esters. Talanta.

[43] Li J., Cai Y., Shi Y., Mou S., Jiang G. (2008). Analysis of phthalates via HPLC-UV in environmental water samples after concentration by solid phase extraction using ionic liquid mixed hemimicelles. Talanta.

[44] Zhang H., Chen X., Jiang X. (2011). Determination of phthalate esters in water samples by ionic liquid cold-induced aggregation dispersive liquid-liquid microextraction coupled with high-performance liquid chromatography. Anal. Chim. Acta.

[45] Yao J., Xu H., Lu L., Songa D., Cui Y., Zhang T., Feng Y.-Q. (2008). A novel liquid-phase microextraction method combined with high-performance liquid chromatography for analysis of phthalate esters in landfill leachates. Anal. Chim. Acta.

[46] Wang X., Song G., Deng C. (2015). Development of magnetic graphene@hydrophilic polydopamine for the enrichment and analysis of phthalates in environmental water samples. Talanta.

[47] Zhang Q., Liu G., Cao X., Yin J., Zhang Z. (2020). Preparation of magnetic zeolitic imidazolate framework-67 composites for the extraction of phthalate esters from environmental water samples. Anal. Methods.

[48] Lirio S., Fu C.-W., Lin Y., Hsu M.J., Huang H.Y. (2016). Solid-phase microextraction of phthalate esters in water sample using different activated carbon-polymer monoliths as adsorbents. Anal. Chim. Acta.

[49] Makkliang F., Kanatharana P., Thavarungkul P., Thammakhet C. (2015). Development of magnetic micro-solid phase extraction for analysis of phthalate esters in packaged food. Food Chem..

[50] Moazzen M., Khaneghah A.M., Shariatifar N., Ahmadloo M., Baghani A.N., Yousefinejad S., Alimohammadi M., Azari A., Dobaradaran S., Rastkari N. (2019). Multi-walled carbon nanotubes modified with iron oxide and silver nanoparticles (MWCNT-Fe_3_O_4_/Ag) as a novel adsorbent for determining PAEs in carbonated soft drinks using magnetic SPE-GC/MS method. Arab. J. Chem..

[51] Bandforuzi S.R., Hadjmohammadi M.R. (2018). Application of non-ionic surfactant as a developed method for the enhancement of two-phase solvent bar microextraction for the simultaneous determination of three phthalates esters from water samples. J. Chromatogr. A.

